# Erratum for Nachman et al., “Increased fungal burden in the gastrointestinal tract of brain-dead organ donors”

**DOI:** 10.1128/spectrum.02858-25

**Published:** 2025-11-25

**Authors:** Erika J. Nachman, Colleen K. Ardis, A. Kyle B. Ardis, Javier Nieto, Madeline M. Bresson, Clare M. Robertson, Maggie N. Seale, Nora M. Villafuerte, Zhe Lyu, Eva C. Preisner, Heather A. Danhof, Sara C. Di Rienzi, Yolanda T. Becker, Robert A. Britton

**Affiliations:** 1 Department of Molecular Virology and Microbiology, Baylor College of Medicine 3989, Houston, Texas, USA; 2 LifeGift, Houston, Texas, USA; 3 Alkek Center for Metagenomics and Microbiome Research, Baylor College of Medicine 661296, Houston, Texas, USA

## ERRATUM

Volume 13, no. 8, e03341-24, 2025, https://doi.org/10.1128/spectrum.03341-24. The affiliations, affiliation numbers, and present addresses for each author should appear as shown in this erratum.

