# Supplementary figures and images for: Increased fungal burden in the gastrointestinal tract of brain-dead organ donors
Source: Microbiol Spectr. 2025 Jun 18;13(8):e03341-24. doi: 10.1128/spectrum.03341-24 (PMC12323666; doi:10.1128/spectrum.03341-24)

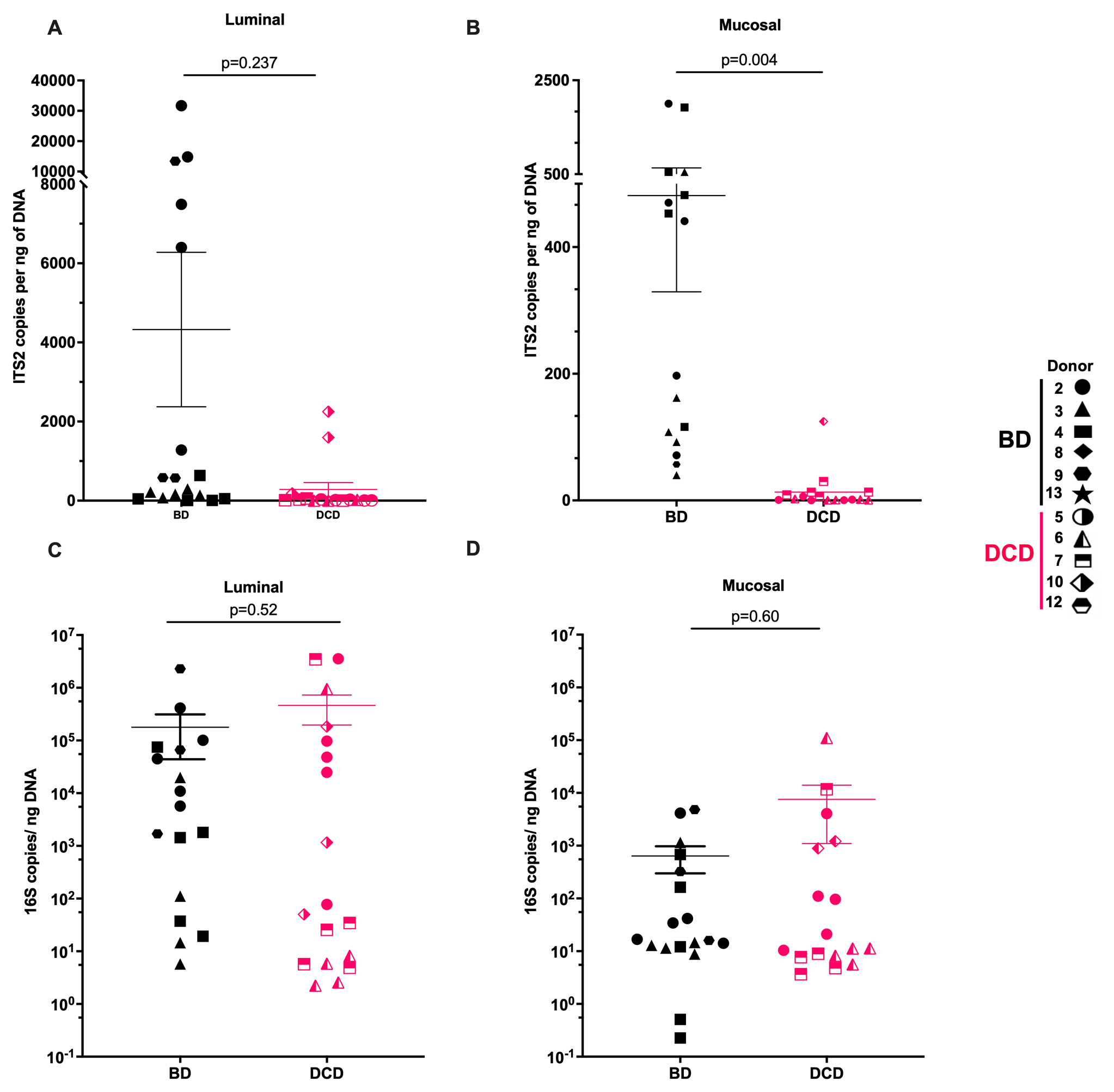

Supplement: Figure S1 — qPCR data for fungal load. [file spectrum.03341-24-s0001.tiff]

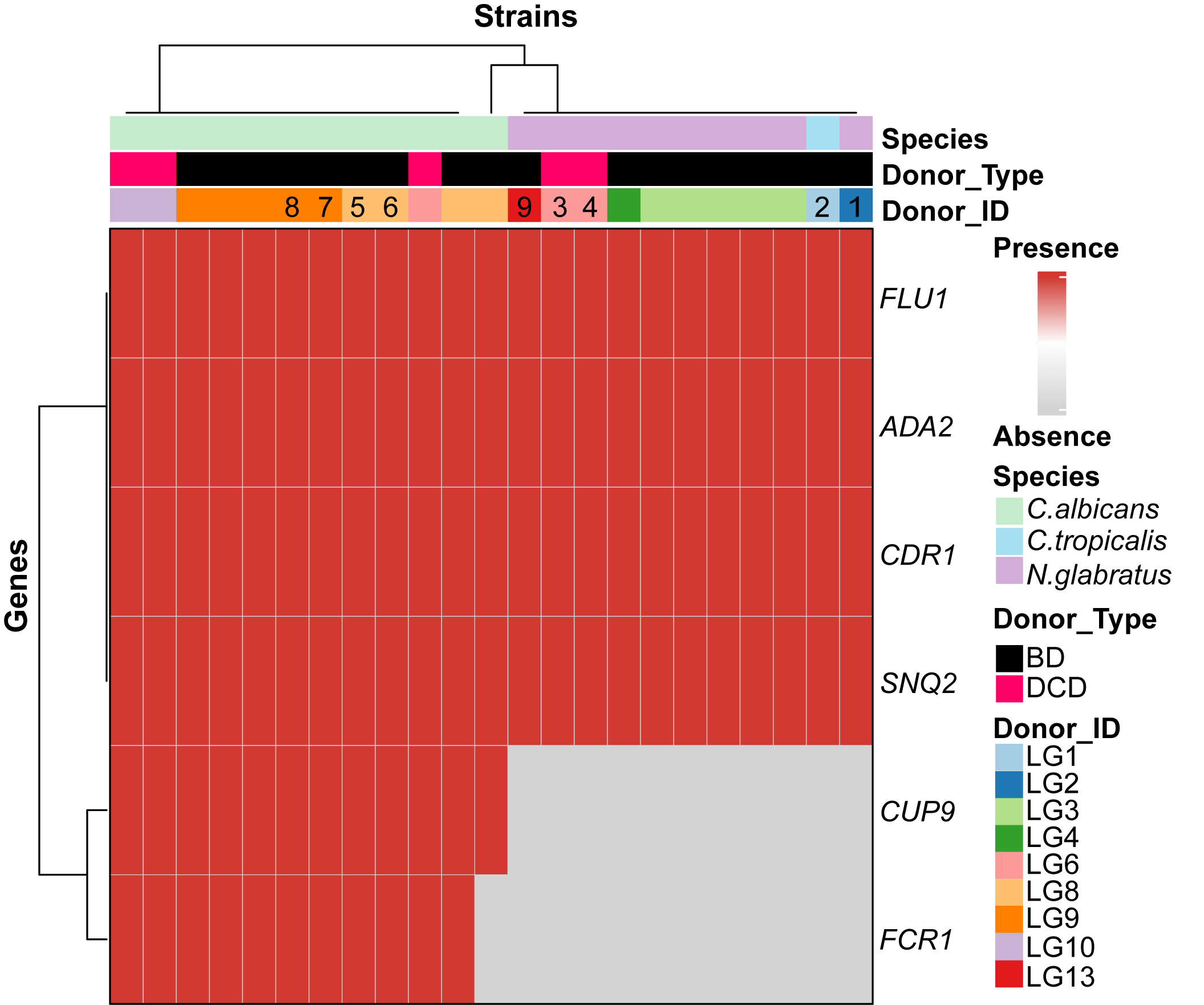

Supplement: Figure S2 — Presence of anti-fungal genes in isolates. [file spectrum.03341-24-s0002.tif]
