# Supplementary material for: Increased fungal burden in the gastrointestinal tract of brain-dead organ donors
Source: Microbiol Spectr. 2025 Jun 18;13(8):e03341-24. doi: 10.1128/spectrum.03341-24 (PMC12323666; doi:10.1128/spectrum.03341-24)
Supplement: Supplemental materials — Tables S1 to S4, supplemental figure legends, and additional methods. [file spectrum.03341-24-s0004.docx]

**Supplementary Information**

**Supplemental Table 1. Extended Metadata on Donors.** We were provided extended information on the donors, such as blood type, body mass index, and cause of death.

| **Donor** | **Type of Donation** | **Blood Type** | **Body Mass Index** | **Cause of Death** |
| --- | --- | --- | --- | --- |
| **2** | BD | AB | 23.2 | Anoxia secondary to drug intoxication overdose |
| **3** | BD | O | 44.4 | Anoxia secondary to cardiac arrest |
| **4** | BD | O | 33.7 | Anoxia secondary to drug intoxication overdose |
| **8** | BD | O | 43.8 | Myocardial infarction |
| **9** | BD | O | 26.9 | Anoxia secondary to suicide |
| **13** | BD | O | 28 | CVA/Stroke |
| **5** | DCD | A1 | 39.2 | Subarachnoid hemorrhage |
| **6** | DCD | B | 37.4 | Gunshot wound to the neck |
| **7** | DCD | A | 23.5 | Motor Vehicle Collision |
| **10** | DCD | A | 29 | CVA/Stroke |
| **12** | DCD | O | 29.6 | Head Trauma |

**Supplemental Table 2. Drug Resistance genes of interest.**

| **Gene** | **Function** | **Drug** |
| --- | --- | --- |
| ***FLU1^21^*** | Multi-drug resistance transporter | Fluconazole |
| ***CDR1^22^*** | Multi-drug resistance efflux pump | Azoles |
| ***ADA2^23^*** | Transcriptional adapter | Azoles, echinocandins, and polyenes |
| ***CUP9^24^*** | Transcription regulator | Caspofungin |
| ***SNQ2^25^*** | Multidrug resistance transporter | Multidrug resistance transporter |
| ***FCR1^26^*** | Fluconazole resistance transcription factor | Fluconazole |

**Supplemental Table 3. Virulence genes of interest function**.

| **Gene(s)** | **Function** |
| --- | --- |
| ***SSA1^27^*** | Invasin |
| ***AWP1^28^*** | Adherence to cell surface |
| ***YAP1^29^, SKN7^29^, MSN4^29^, HOG1^30^*** | Oxidative Stress, Macrophage Survival |
| ***VPS34^31^*** | Macrophage survival  phosphatidylinositol 3’-kinase |
| ***HWP1^32^*** | Transglutaminase adhesionhyphal specific gene |
| ***ALS1, ALS3 ^33,34^*** | Adherence to cell surface |
| ***ECE1^37^*** | Candidalysin |
| ***SAP4, SAP5^36^*** | Aspartyl protease |
| ***LIP8^37^*** | Lipase |
| ***EFG1^38^*** | Hyphal formation |

**Supplemental Table 4. Isolates from whole genome sequencing metadata.** The isolates that underwent whole genome sequencing are described based on species, donor type, and donor ID. All were isolated from the gastrointestinal tract (n=23).

| Isolate | Species | Donor_Type | Donor_ID | Name_on_figure |
| --- | --- | --- | --- | --- |
| 1 | *C.tropicalis* | BD | LG2 | *C. tropicalis*, donor 1 |
| 2 | *N.glabratus* | BD | LG1 | *N. glabratus*, donor 1 |
| 3 | *N.glabratus* | BD | LG3 |  |
| 4 | *N.glabratus* | BD | LG3 |  |
| 5 | *N.glabratus* | BD | LG3 |  |
| 6 | *N.glabratus* | BD | LG3 |  |
| 7 | *N.glabratus* | BD | LG3 |  |
| 8 | *N.glabratus* | BD | LG4 |  |
| 9 | *N.glabratus* | DCD | LG6 | *N. glabratus*, donor 6, A |
| 10 | *N.glabratus* | DCD | LG6 | *N. glabratus*, donor 6, B |
| 11 | *N.glabratus* | BD | LG13 |  |
| 12 | *C.albicans* | DCD | LG6 |  |
| 13 | *C.albicans* | BD | LG8 |  |
| 14 | *C.albicans* | BD | LG8 | *C albicans*, donor 8, A |
| 15 | *C.albicans* | BD | LG8 | *C albicans*, donor 8, B |
| 16 | *C.albicans* | BD | LG8 |  |
| 17 | *C.albicans* | BD | LG9 |  |
| 18 | *C.albicans* | BD | LG9 |  |
| 19 | *C.albicans* | BD | LG9 | *C albicans*, donor 9, A |
| 20 | *C.albicans* | BD | LG9 |  |
| 21 | *C.albicans* | BD | LG9 | *C albicans*, donor 9, B |
| 22 | *C.albicans* | DCD | LG10 | *C albicans*, donor 10 |
| 23 | *C.albicans* | DCD | LG10 |  |

qPCR methods

**Fungi Quant**

- 2X mastermix: Quanta Bio PerfeCTa qPCR ToughMIx, low ROX, Cycling conditions: 10 min (95°C) +  [40 cycles (15 sec, 95°C), (60 sec, 60°C)], Probe: (6FAM) TGG TGC ATG GCC GTT (MGBNFQ)
- 16S
  - 2X mastermix: NEB Luna Universal qPCR mastermix
  - Primers (final conc): 500 nM
  - Suzuki MT, Taylor LT, DeLong EF. 2000. Quantitative Analysis of Small-Subunit rRNA Genes in Mixed Microbial Populations via 5′-Nuclease Assays. Appl Environ Microbiol 66**:.**[**https://doi.org/10.1128/AEM.66.11.4605-4614.2000**](https://doi.org/10.1128/AEM.66.11.4605-4614.2000)

**Supplementary figure legends.**

**Supplementary Figure 1.** Higher fungal genomic content in BD donor samples. Select luminal samples (A) and mucosal samples (B) that were flash-frozen day of collection were quantified using qRT-PCR from the stomach, duodenum, jejunum, ileum, and colon. Select luminal samples (C) and mucosal samples (C) were quantified using qRT-PCR for the 16S rRNA gene from the stomach, duodenum, jejunum, ileum, and colon. ^†^Statistical significance was determined by a mixed linear model, and exact p values are displayed.

**Supplemental Figure 2. Fungal isolates have predicted drug-resistance genes.** Selected genes displayed are as in Supplementary Table 3. Presence and absence are differentiated by red and grey, respectively, with donor type as black (BD) and pink (DCD). Species are colored green (*C. albicans*), blue (*C. troplicalis)*, and purple (*N. glabratus*).

**Supplemental Figure 3. Fungal isolates possess predicted fungal virulence genes.** Selected genes displayed are as in Supplementary Table 3 with all tested samples. Presence and absence are differentiated by red and grey, respectively with donor type as black (BD) and pink (DCD). Species are colored green (*C. albicans*), blue (*C. troplicalis)* (A), and purple (*N. glabratus*) (B).
